# A Comparison of the Effects of Neuronal Nitric Oxide Synthase and Inducible Nitric Oxide Synthase Inhibition on Cartilage Damage

**DOI:** 10.1155/2016/7857345

**Published:** 2016-06-13

**Authors:** Nevzat Selim Gokay, Ibrahim Yilmaz, Baran Komur, Ahu Senem Demiroz, Alper Gokce, Sergülen Dervisoglu, Banu Vural Gokay

**Affiliations:** ^1^Istanbul Esenyurt University, 34510 Istanbul, Turkey; ^2^Department of Pharmacovigilance, Materiovigilance and Rational Use of Drugs, Tekirdag State Hospital, Ministry of Health, 59100 Tekirdag, Turkey; ^3^Kanuni Sultan Suleyman Training and Research Hospital, Turgut Ozal Street No. 1, Halkali, Kucukcekmece, 34303 Istanbul, Turkey; ^4^Istanbul University, Cerrahpasa School of Medicine, Department of Pathology, Istanbul University, 34098 Istanbul, Turkey; ^5^Nisantasi University, 34360 Istanbul, Turkey; ^6^Department of Anesthesiology and Reanimation, Acıbadem University, 34360 Istanbul, Turkey

## Abstract

The objective of this study was to investigate the effects of selective inducible nitric oxide synthase and neuronal nitric oxide synthase inhibitors on cartilage regeneration. The study involved 27 Wistar rats that were divided into five groups. On Day 1, both knees of 3 rats were resected and placed in a formalin solution as a control group. The remaining 24 rats were separated into 4 groups, and their right knees were surgically damaged. Depending on the groups, the rats were injected with intra-articular normal saline solution, neuronal nitric oxide synthase inhibitor 7-nitroindazole (50 mg/kg), inducible nitric oxide synthase inhibitor amino-guanidine (30 mg/kg), or nitric oxide precursor L-arginine (200 mg/kg). After 21 days, the right and left knees of the rats were resected and placed in formalin solution. The samples were histopathologically examined by a blinded evaluator and scored on 8 parameters. Although selective neuronal nitric oxide synthase inhibition exhibited significant (*P* = 0.044) positive effects on cartilage regeneration following cartilage damage, it was determined that inducible nitric oxide synthase inhibition had no statistically significant effect on cartilage regeneration. It was observed that the nitric oxide synthase activation triggered advanced arthrosis symptoms, such as osteophyte formation. The fact that selective neuronal nitric oxide synthase inhibitors were observed to have mitigating effects on the severity of the damage may, in the future, influence the development of new agents to be used in the treatment of cartilage disorders.

## 1. Introduction

Osteoarthritis (OA) is a progressive disorder that involves cartilage loss. The search continues for a medical or surgical treatment for the cartilage damage that is often blamed for triggering the disorder. In recent years, studies aimed at understanding OA pathophysiology have been conducted. Mechanisms that prevent the formation of the disorder and its advance will also advance treatment methods.

Cartilage loss and subchondral bone resorption are known to develop as a result of a catabolic chemical cascade [1]. Cartilage tissue loss and OA are a result of a breakdown in the balance between cartilage extracellular matrix synthesis and degradation in the catabolic direction [2]. Cytokines that stimulate matrix proteinases (MMP) contribute to the catabolic process [3]. These cytokines also trigger the formation of nitric oxide (NO) in the joints [4]. It has been reported that NO causes cartilage degradation by increasing the effect of IL-1 and triggering apoptosis [5]. NO, which is formed by the oxidation of the guanidino nitrogen of L-arginine, is synthesized by different NO synthases (NOS) in neuronal, endothelial, and inducible manners [6]. It has been shown that inducible NOS (iNOS) is found more often in cells with OA compared to normal cells [6–9]. The inhibition of iNOS decreased the loss of glycosaminoglycan content in an OA model [10]. However, a recent prospective clinical study on OA patients reported that iNOS inhibition had no effect on OA progression [11]. The protective effects of NO were also emphasized in a recent review about NO and OA [12]. It has been thought that neuronal NOS (nNOS) might play a more dominant role in the development of the disorder [13]. nNOS activity has been found to be increased in human chondrocytes with OA compared with normal chondrocytes [14]. Few histopathological studies have investigated whether nNOS or iNOS play a more dominant role in the etiopathogenesis of cartilage damage.

From a histopathological perspective, our objective in this study was to investigate and to compare the effectiveness of select nNOS and iNOS inhibitors on cartilage damage in Wistar-type male rats to treat experimentally induced joint damage.

## 2. Method

This study was performed with a live mammal use permit granted by the T.R. Namik Kemal University Experimental Animals Local Ethical Board (Meeting Decision Number 2010/04, dated 01.06.2010), which follows the guidelines of the Turkish Animal Experimentation Regulations. The experimental analyses were repeated 3 times.

### 2.1. Materials

Wistar-type male rats were obtained from Istanbul University (Experimental Medicine Research Institute, Vakif Gureba Caddesi, 34093 Capa, Istanbul); 7-nitroindazole (N7778-5G), amino-guanidine, and L-arginine were obtained from Sigma-Aldrich Chemie GmbH (Steinheim, Germany), and 0.9% NaCl (saline solution) was obtained from Biofarma Drug Industry and Commerce, Inc. (Istanbul, Turkey).

### 2.2. Methods

A total of 27 Wistar-type male rats weighing an average 300 g (240–350 g) and with an average age of eighteen weeks were used. All efforts were made to minimize animal suffering and the number of animals used. Experimental Study Design, which is discussed in the National Center for the Replacement, Refinement & Reduction of Animals in Research (http://www.nc3rs.org.uk/experimental-design), guided us in determining groups and numbers. The animals were granted ad libitum access to food and water and were maintained on a 12-hour light and dark cycle. The rats were randomly separated into 5 groups, with 3 rats in the first group and 6 in each of the other four groups. Rats were kept in different cages in each group at 22.4°C. All of the procedures were performed in the T.R. Namik Kemal University Experimental Animal Laboratory.

On Day 1 of the experiment, the right and left knees of the 3 rats in the first group were resected, without dissecting the muscles or opening the capsule, under anesthesia with administration of an overdose of intraperitoneal sodium pentobarbital. The knees were placed in 10% formaldehyde and used as the control group (Group 1, *n* = 6) (22.4°C under normal conditions (UNC)). The rats in Group 1 were sacrificed by cervical dislocation after this procedure. Arthrotomy was performed under anesthesia on the right knees of the remaining 24 rats in 4 groups (Groups 2, 3, 4, and 5; *n* = 6 in each group), and a full-thickness osteochondral defect was created in the medial condyle.

**Figure 1:**
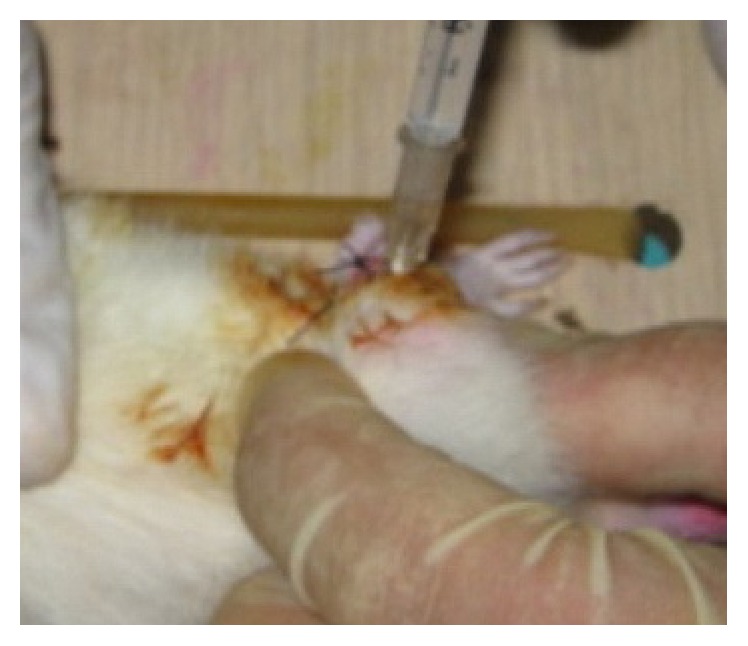
Using an insulin injector, active ingredients were injected into the right knees of the rats according to the group.

On Day 2 of the experiment, the injections were started, and for 7 days, pharmacological ingredients that were prepared based on the groups were injected twice daily into the defect generated in the right knees of the rats (morning and evening, Table S1 in Supplementary Material available online at http://dx.doi.org/10.1155/2016/7857345). Each time, the same sequence from Group 2 to Group 5 was followed during injections. An insulin injector (100 IU) was used, and the injection was performed into the joint through the medial patellar tendon while the knee was at 90° flexion (Figure 1). The needle's entry into the joint space was checked by the loss of resistance to the needle.

On Day 29 of the experiment, the defective right knees and the intact left knees of all rats (to determine the systemic effects of the injected active ingredients) were resected without dissecting the muscles or opening the capsules, under anesthesia with administration of an overdose of intraperitoneal sodium pentobarbital. All of the samples were preserved in 10% formaldehyde (22.4°C UNC). Then, the rats were sacrificed by cervical dislocation.

The obtained materials were coded and sent to the Pathology Laboratory of Istanbul University's Cerrahpaşa Medical School for histopathological evaluation.

### 2.3. Applied Surgical Technique

**Figure 2:**
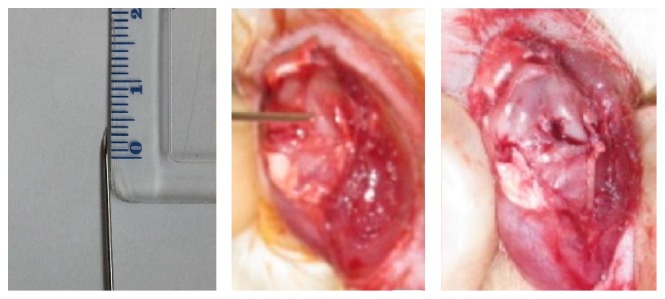
Full-thickness cartilage defects were generated with a 21-gauge needle.

All surgical procedures were performed under aseptic conditions by a single surgeon (NSG). The rats were anesthetized with an intraperitoneal administration of 30 mg/kg sodium pentobarbital, and their right knees were shaved. Entry to the rats' right knees was through an anterior skin incision. The capsule was opened using a medial parapatellar approach, and care was taken to prevent iatrogenic joint injury. A full-thickness osteochondral defect (0.8 mm thickness × 4 mm depth) was generated in the medial condyle with the tip of an injection needle (21-gauge sterile needle, Beybi Plastik Fab. San. AŞ., Istanbul, Turkey) [15–17]. Lesions were created with a 21-gauge needle (0.8 mm in diameter, 4 mm bevel length) to the lateral wall of the medial condyle at the level of the intersection of the trochlear groove and the intercondylar notch (Figure 2). The needle was inserted in one attempt, until the bevel of the needle was entirely inside the femur. The skin was closed and the procedure ended.

### 2.4. Histopathological Evaluation

After routine tissue processing, the tissue samples were embedded in paraffin blocks. Sections of 5 *μ*m thickness were stained with hematoxylin-eosin and examined with a light microscope. The pathologist who performed the examinations was blinded to the experimental groups, drugs used, and which knee joint was being examined. The histological sections were evaluated and scored based on the condition of the surface layer, clone formation of the chondrocytes, single cell death in the chondrocytes, decreases in cartilage layer thickness, increases in subchondral bone thickness, chronic synovitis findings, and synovial cyst and osteophyte formations (Table S2) [18].

### 2.5. Statistical Evaluation

SPSS 22.0 (IBM statistics for Windows version 22, IBM Corporation, Armonk, New York, United States) was used to analyze the data. The Mann-Whitney *U* Test was used to compare two independent samples. The Kruskal-Wallis *H* Test was used to compare between multiple groups, and the nonparametric Post hoc Test (Miller 1966) was used for post hoc analyses. Pearson chi-square, linear-by-linear association, and Fisher exact tests were tested using the Monte Carlo simulation technique to compare categorical data. The Monte Carlo simulation technique results were used in all analyses. Quantitative data are given in tables as the mean ± SD (standard deviation), median ± IQR (interquartile range), and median and range (maximum–minimum). Categorical data are given as *n* (numbers) and percentages (%). The data were analyzed in 95% confidence intervals, and statistical significance was set at *P* < 0.05.

## 3. Results

No complications were encountered during the surgeries, injections, and follow-up period. The postoperative behavior of all rats was no different than that observed preoperatively, with normal feeding and behavior towards handlers.

As mentioned in methods section, both knees were resected without any defects on Day 1 of the experiment in Group 1, intra-articular SF (15 mg/kg/day) was applied to the defect-induced right knees for 7 days in Group 2, intra-articular SF (15 mg/kg/day) + 7-nitroindazole (50 mg/kg/day) was applied to the defect-induced right knees for 7 days in Group 3, intra-articular SF (15 mg/kg/day) + amino-guanidine (30 mg/kg/day) was applied to the defect-induced right knees for 7 days in Group 4, and intra-articular SF (15 mg/kg/day) + L-arginine (200 mg/kg/day) was applied to the defect-induced right knees for 7 days in Group 5 (Table S1).

Comparing the right and left knees of Group 2 to Group 5, there was statistically significantly worse results in right knees of Group 5, according to all parameters (Table S3). There were no statistically significant differences between the right and left knees of Group 3 and Group 4 according to chronic synovitis parameters (*P* = 0.316 and *P* = 0.235, resp.). According to surface characteristics, there were statistically significantly worse results in the right knees of each group (*P* = 0.002).

**Figure 3:**
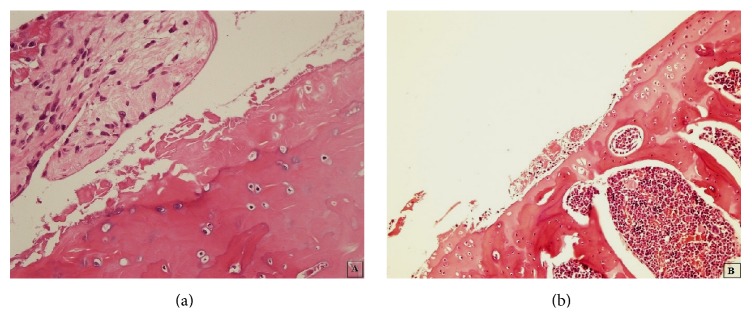
Predominant fissuration ((a) a histological section taken from the right knee of a rat in Group 3, HE ×400) and erosion ((b) a histological section taken from the right knee of a rat in Group 3, HE ×100) were observed in the right (defective) knees of the rats in Group 3.

**Figure 4 fig4:**
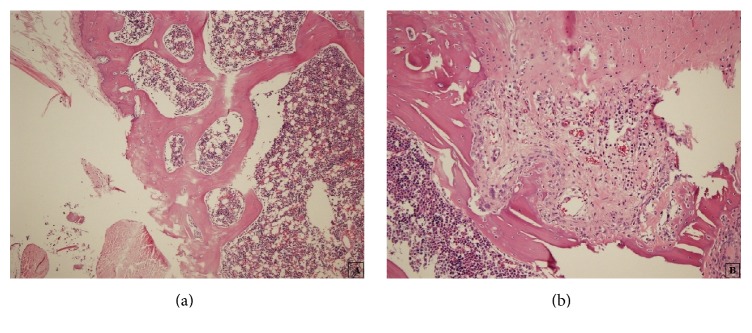
Predominant denudation ((a) a histological section from the right knee of a rat from Group 4, HE ×100) and deformation ((b) a histological section from the right knee of a rat from Group 5, HE ×200) were observed in the right (defective) knees of the rats from Groups 4 and 5.

**Figure 5 fig5:**
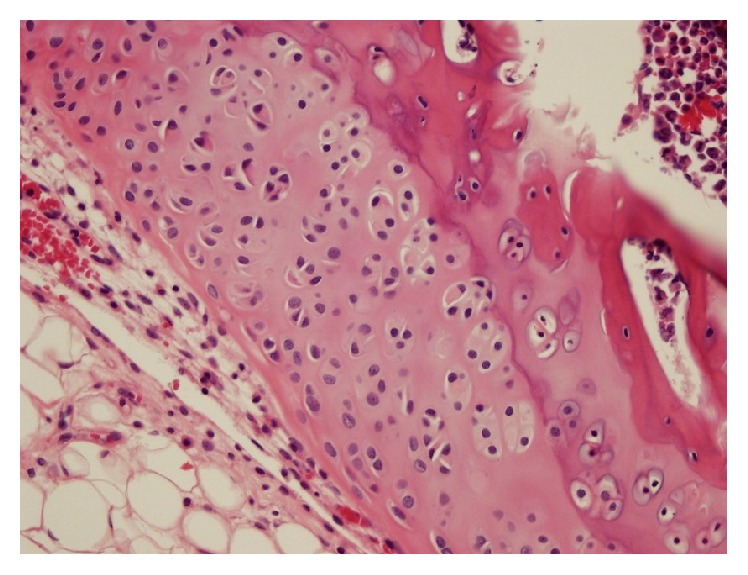
A histological section taken from the right knee of a rat from Group 4 shows large clones consisting of large numbers of chondrocytes (HE ×400).

**Figure 6:**
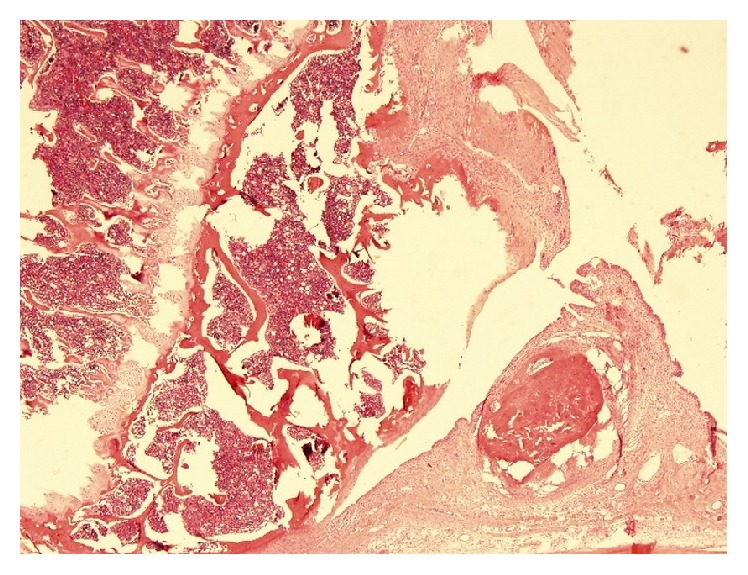
A histological section taken from the right knee of a rat from Group 5 shows the development of an osteophyte on the right bottom (HE ×100).

There were significantly different results observed for each parameter when Group 1 knees and the right knees of all other groups were compared (Table S4). While there was no clone formation in Group 1, there were 2 small clones and 4 large clones in Group 5. Large clones were mostly observed in Group 5 compared with 2 in Group 2, 0 in Group 3, and 3 in Group 4. Osteophyte formation was also only observed in Group 5 (*P* = 0.003) (Figures 3 [Fig fig4] [Fig fig5]–6).

**Figure 7:**
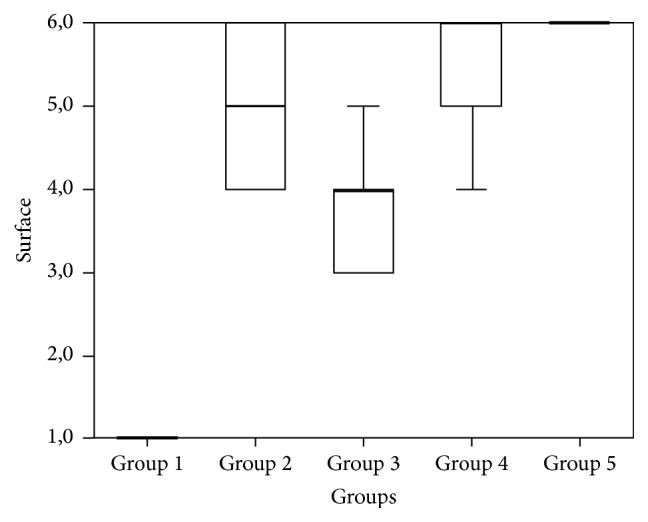
A surface characteristics distribution graph for Group 1 and the right (defective) knees of all other groups (Groups 2–5).

In Groups 2, 3, 4, and 5, statistically significant worse surface characteristic results were observed compared to those in Group 1 (*P* < 0.001). Statistically significant worse results were found compared to those in Group 3 (*P* < 0.001) in Groups 4 and 5 (Table S4, Figure 7).

## 4. Discussion

In this study, we formed a mechanical OA model in Wistar rats and then attempted to cure the pathology with different agents. The histopathologic evaluations were blindly performed by a pathologist who is an expert in musculoskeletal pathology. There are a number of limitations in this study. The chosen species may be a bit too small to indicate that the results are also appropriate for humans. We chose this species because of their advantages in terms of cost and housing. Additionally, there are rat OA models used to study the treatment of OA in the literature [19]. The surgical technique we used to form an OA model was recently published [17]. The placement of defect lesions was guided by human approximation. Needle punching was performed in a single attempt, and care was taken to improve standardization. The homogeneous results observed within the groups helped us to overcome this bias. The histopathologic evaluation and scoring of the severity of cartilage damage were performed by the same observer with an individual and previously published scoring system [18]. The system has some alterations other than Osteoarthritis Research Society International (OARSi) recommendations but evaluates the same topics, surface characteristics, osteophyte formation, synovial changes, and cartilage and bone thickness [20]. The scoring system used in this study depends on yes or no answers; therefore, this system seems more objective for standardization to us. Furthermore, another rationale for us to use this scoring system is that it is the system with which our observer is experienced.

OA is a disorder that involves progressive deterioration and cartilage loss and is characterized by the final disruption of joint functions. This deterioration of the cartilage is triggered by the synthesis of some catabolic substances in the diarthrodial joints. MMP and NO, which are thought to play important roles in the catabolic process, are released through the stimulation of cytokines, such as IL-1 and TNF-*α*, and NOS [21].

NO is synthesized from L-arginine amino acids by 2 different NOS that are referred to by the name of the cells in which they exist. In vascular cells, NOS is called endothelial NOS (eNOS), and in nerve cells, it is called nNOS. In addition to their systemic effects, it has been reported that both NOS play roles in the proliferation of the cartilage tissue during its development [22, 23]. In addition, the existence of a third NOS, iNOS, which is not normally present in cells but is induced as a result of the secreted cytokines, has been discovered [24, 25]. Similar to the others, iNOS uses the primary substance of L-arginine amino acid in NO synthesis and is thought to be responsible for the tissue breakdown in various disorders, including cartilage breakdown in OA.

In inflammatory disorders, many proinflammatory cytokines and iNOS are secreted from the synovial membrane, but in the case of OA, they are expressed by the affected cartilage cells more than the synovial membrane [26]. Furthermore, it has been shown that the isolated cartilage cultures obtained from OA patients synthesized greater amounts of NO compared with cartilage cultures obtained from patients with rheumatoid arthritis [27]. This condition has been attributed to an excess production of cytokines in cartilage cells with OA and the upregulation of NOS [4, 6, 27–30]. Mechanical stress has also been shown to increase the production of NO from chondrocytes [31]. More NO is secreted from arthritic cartilage tissue compared to normal cartilage tissue [6, 32].

Although the literature has demonstrated that iNOS and NO are found in increased amounts in cartilage cells with OA, there is not enough information on the role NO plays in the development of the disorder. The fact that NO suppresses the synthesis of the IL-1 receptor antagonist and increases the effectiveness of IL-1 results in it being blamed for the pathogenesis [33]. Furthermore, it is believed that NO's activation of matrix metalloproteinases also plays a role in the pathogenesis [34]. In addition, it is also believed that, in an indirect manner, NO contributes to the catabolic process by increasing the expression of cytokines, such as IL-18 and IL-1 [35]. NO has also been reported to trigger cell death [36]. Interestingly, our study observed cell death in the samples in which we had inhibited NO with iNOS and nNOS and also in those in which we increased NO, which was significantly greater compared with that in the right knees of the rats in Group 2 (*P* < 0.001).

In some studies that attempted to suppress the iNOS gene, it was concluded that iNOS does not play an indicative role in the course of OA; in fact, it exhibits a prophylactic effect on the cartilage by increasing the synthesis of proteoglycan and decreasing breakdown [37, 38]. Tamura et al. stated that basic fibroblast growth factor is secreted into the environment through the influence of NO, and as a result, NO has indirect favorable effects on cell proliferation and angiogenesis [39]. These studies indicate that NO might be a substance synthesized by activating the iNOS pathway after damage to the joint to protect the cartilage and to increase its regeneration. Häuselmann et al. determined that different cartilage layers synthesize different amounts of NO in response to IL-1 stimulation and that different layers react differently to NOS inhibition [40]. The authors showed that N^G^-monomethyl-L-arginine (L-NMA) completely nullifies the inhibitory effect of IL-1 on proteoglycan in the deeper layers of the cartilage. They also determined that the stimulatory effect of IL-1 on NO decreases with age. The authors believe that, based on these results, NO might play a prophylactic role in the proteoglycan catabolism. Bezerra et al. disclosed that, in an OA model created with zymosan, selective iNOS inhibitors or nonselective NOS inhibitors reduced inflammation but did not affect the glycosaminoglycan loss [41]. In support of these findings, it was also observed in our study that there was no statistically significant difference in surface characteristics in the samples in which iNOS was blocked and NO was induced (*P* < 0.001).

These findings show that the role of NO in the joint is more complicated than previously assumed, and more studies are needed to fully understand its efficacy. The majority of studies on NO and OA have been based on the selective inhibition of iNOS, the secretion of which has been found to increase in cartilage with OA. Because the protective effect of iNOS on joint cartilage has been determined in some studies, the question remains whether the other NOSs may have a predominant effect on OA etiopathogenesis. In a study investigating the effects of nNOS inhibition on endochondral bone development, a decrease in the proliferation of chondrocytes upon the blockage of the nNOS gene was reported [23]. Pozza et al. investigated the role of the peripheral nervous system in OA and disclosed that neuronal NO affects the development of the disorder [13].

There are some studies which evaluate NO role in immunologic pathways. Paolucci et al. investigated NO inhibition in TNF-alpha regulation in endocytosis of human dendritic cell [42]. They found antagonist effect with TNF-alpha and NO. In another study of Falcone et al., they investigated protective effect of NO in apoptosis of dendritic cells [43]. These kinds of studies also refer to protective effect of NO in cellular mechanism.

Our study demonstrated that the suppression of NO synthesis by iNOS in rats with an experimental cartilage defect did not affect cartilage regeneration. However, a distinct improvement was observed in the surface characteristics in the knees of the rats in which the synthesis of NO was suppressed with nNOS. Deterioration in all pathological parameters was observed in the group in which NO synthesis was induced, and unlike other groups, osteophytes developed in the knees of the rats in this group.

## 5. Conclusion

These findings show that different NOSs affect cartilage metabolism differently. In our study, it was histopathologically shown that, similar to the results obtained by Pozza et al. [13], nNOS inhibition has positive effects on cartilage healing, whereas iNOS inhibition has no significant effect. These findings may provide guidance for the development of new methods to treat OA in the future. New studies are needed to investigate the influence of different NOSs in NO synthesis and OA etiopathogenesis.

## Supplementary Material

Treatment methods according to groups is summarized in this Table. We applied Intra-articular SF, 7-nitroindazole and amino-guanidine to different groups to investigate the effect.
