# Supplementary material for: A Comparison of the Effects of Neuronal Nitric Oxide Synthase and Inducible Nitric Oxide Synthase Inhibition on Cartilage Damage
Source: Biomed Res Int. 2016 Jun 13;2016:7857345. doi: 10.1155/2016/7857345 (PMC4921627; doi:10.1155/2016/7857345)
Supplement: Supplementary file 1 — Treatment methods according to groups is summarized in this Table. We applied Intra-articular SF, 7-nitroindazole and amino-guanidine to different groups to investigate the effect. [file 7857345.f1.doc]

**Table S1.** Treatment methods according to groups

| Group 1 (n=6) | Both knees were resected without any defects on day 1 of the experiment. |
| --- | --- |
| Group 2 (n=6) | Intra-articular SF (15 mg/kg/day) was applied to the defect-induced right knees for 7 days. |
| Group 3 (n=6) | Intra-articular SF (15 mg/kg/day) + 7-nitroindazole (50 mg/kg/day) was applied to the defect-induced right knees for 7 days. |
| Group 4 (n=6) | Intra-articular SF (15 mg/kg/day) + amino-guanidine (30 mg/kg/day) was applied to the defect-induced right knees for 7 days. |
| Group 5 (n=6) | Intra-articular SF (15 mg/kg/day) + L-arginine (200 mg/kg/day) was applied to the defect-induced right knees for 7 days. |

**Table S2.** Histopathological evaluation parameters and scoring

| **1-Condition of the surface layer** | | | | | | | | |
| --- | --- | --- | --- | --- | --- | --- | --- | --- |
| **Score** | 1 | 2 | | 3 | | 4 | 5 | 6 |
| **Definition** | Intact | Fibrillation | | Fissuration | | Erosion | Denudation | Deformation |
| **2- Clone formation of chondrocytes** | | | | | | | | |
| **Score** | 0 | | 1 | | | | 2 | |
| **Definition** | No clone formation | | Small clones made up of 0-3 chondrocytes are present | | | | Large clones made up of 4 or more chondrocytes are present | |
| **3- Single cell death in chondrocytes** | | | | | | | | |
| **Score** | 0 | | | | 1 | | | |
| **Definition** | No | | | | Yes | | | |
| **4- Decrease in cartilage layer thickness** | | | | | | | | |
| **Score** | 0 | | | | 1 | | | |
| **Definition** | No | | | | Yes | | | |
| **5- Increase in subchondral bone thickness** | | | | | | | | |
| **Score** | 0 | | | | 1 | | | |
| **Definition** | No | | | | Yes | | | |
| **6- Chronic synovitis findings** | | | | | | | | |
| **Score** | 0 | | 1 | | | | 2 | |
| **Definition** | No | | Mild findings of synovitis | | | | Severe findings of synovitis | |
| **7- Synovial cyst formation** | | | | | | | | |
| **Score** | 0 | | | | 1 | | | |
| **Definition** | No | | | | Yes | | | |
| **8- Osteophyte formation** | | | | | | | | |
| **Score** | 0 | | | | 1 | | | |
| **Definition** | No | | | | Yes | | | |

**Table S3.** Comparison of the right (defective) and left (intact) knees of Group 2, Group 3, Group 4 and Group 5

|  | | **GROUP 2** | **GROUP 3** | **GROUP 4** | **GROUP 5** |
| --- | --- | --- | --- | --- | --- |
|  |  | Right / Left | Right / Left | Right / Left | Right / Left |
| **SURFACE** | |  | | | |
| Median(Max-Min) | | 5(6-4) / 1(2-1) | 4(5-3) / 1(2-1) | 6(6-4) / 1(1-1) | 6(6-5) / 1(1-1) |
| **P Value** | | **0.002** | **0.002** | **0.002** | **0.002** |
| **CLONE FORMATION** | No | 0(0) / 1(16,7) | 0(0) / 1(16,7) | 0(0) / 5(83,3) | 0(0) / 0(0) |
|  | SC | 4(66,7) / 5(83,3) | 6(100) / 5(83,3) | 3(50) / 1(16,7) | 2(33,3) / 6(100) |
|  | LC | 2(33,3) / 0(0) | 0(0) / 0(0) | 3(50) / 0(0) | 4(66,7) / 0(0) |
| **P Value** | | **0.279** | **0.317** | **0.008** | **0.019** |
| **SINGLE CELL DEATH** | No | 4(66,7) / 6(100) | 1(16,7) / 6(100) | 0(0) / 6(100) | 0(0) / 4(66,7) |
|  | Yes | 2(33,3) / 0(0) | 5(83,3) / 0(0) | 6(100) / 0(0) | 6(100) / 2(33,3) |
| **P Value** | | **0.455** | **0.015** | **0.002** | **0.006** |
| **DECREASE IN CT** | No | 1(16,7) / 4(66,7) | 1(16,7) / 6(100) | 0(0) / 6(100) | 1(16,7) / 5(83,3) |
|  | Yes | 5(83,3) / 2(33,3) | 5(83,3) / 0(0) | 6(100) / 0(0) | 5(83,3) / 1(16,7) |
| **P Value** | | **0.242** | **0.015** | **0.002** | **0.016** |
| **INCREASE IN BT** | No | 1(16,7) / 4(66,7) | 0(0) / 6(100) | 0(0) / 6(100) | 1(16,7) / 6(100) |
|  | Yes | 5(83,3) / 2(33,3) | 6(100) / 0(0) | 6(100) / 0(0) | 5(83,3) / 0(0) |
| **P Value** | | **0.242** | **0.002** | **0.002** | **0.015** |
| **CHRONIC SYNOVITIS** | No | 2(33,3) / 6(100) | 2(33,3) / 5(83,3) | 2(33,3) / 5(83,3) | 0(0) / 4(66,7) |
|  | Mild | 4(66,7) / 0(0) | 2(33,3) / 1(16,7) | 3(50) / 1(16,7) | 1(16,7) / 2(33,3) |
|  | Severe | 0(0) / 0(0) | 2(33,3) / 0(0) | 1(16,7) / 0(0) | 5(83,3) / 0(0) |
| **P Value** | | **0.006** | **0.316** | **0.235** | **0.008** |
| **SYNOVIAL CYST** | No | 4(66,7) / 6(100) | 4(66,7) / 6(100) | 1(16,7) / 6(100) | 3(50) / 6(100) |
|  | Yes | 2(33,3) / 0(0) | 2(33,3) / 0(0) | 5(83,3) / 0(0) | 3(50) / 0(0) |
| **P Value** | | **0.455** | **0.455** | **0.015** | **0.023** |
| **OSTEOPHYTE** | No | 6(100) / 6(100) | 6(100) / 6(100) | 6(100) / 6(100) | 2(33,3) / 6(100) |
|  | Yes | 0(0) / 0(0) | 0(0) / 0(0) | 0(0) / 0(0) | 4(66,7) / 0(0) |
| **P Value** | | **-** | **-** | **-** | **0.006** |

*Mann Whitney U Test - Pearson Chi Square Test - Likelihood Ratio - Fisher's Exact Test - Linear-by-Linear AssociationMonte Carlo Simulation results were used in tests*

*n (%), Max.: Maximum, Min: Minimum, SC: Small Clones, LC: Large Clones, CT: Cartilage Thickness, BT: Bone Thickness*

**Table S4.** Comparison of Group 1 knees and right knees of Groups 2, 3, 4 and 5

|  | **GROUP 1** | **GROUP 2** | **GROUP 3** | **GROUP 4** | **GROUP 5** | **P Value** |
| --- | --- | --- | --- | --- | --- | --- |
| **SURFACE** |  | | | | | **<0.001** |
| Median(Max-Min) | 1 (1-1) | 5(6-4) **ᵃ** | 4(5-3)**ᵃ** | 6(6-4) **ᵃ ͨ** | 6(6-5) **ᵃ ͨ** |
| **CLONE FORMATION** |  | | | | | **<0.001** |
| No / SC / LC | 6(100) / 0(0) / 0(0) | 0(0) / 4(66,7) / 2(33,3) | 0(0) / 6(100) / 0(0) | 0(0) / 3(50) / 3(50) | 0(0) / 2(33,3) / 4(66,7) |
| **SINGLE CELL DEATH** |  | | | | | **<0.001** |
| No / Yes | 6(100) / 0(0) | 4(66,7) / 2(33,3) | 1(16,7) / 5(83,3) | 0(0) / 6(100) | 0(0) / 6(100) |
| **DECREASE IN CT** |  | | | | | **0.001** |
| No / Yes | 6(100) / 0(0) | 1(16,7) / 5(83,3) | 1(16,7) / 5(83,3) | 0(0) / 6(100) | 1(16,7) / 5(83,3) |
| **INCREASE IN BT** |  | | | | | **<0.001** |
| No / Yes | 6(100) / 0(0) | 1(16,7) / 5(83,3) | 0(0) / 6(100) | 0(0) / 6(100) | 1(16,7) / 5(83,3) |
| **CHRONIC SYNOVITIS** |  | | | | | **0.002** |
| No / Mild / Severe | 6(100) / 0(0) / 0(0) | 2(33,3) / 4(66,7) / 0(0) | 2(33,3) / 2(33,3) / 2(33,3) | 2(33,3) / 3(50) / 1(16,7) | 0(0) / 1(16,7) / 5(83,3) |
| **SYNOVIAL CYST** |  | | | | | **0.024** |
| No / Yes | 6(100) / 0(0) | 4(66,7) / 2(33,3) | 4(66,7) / 2(33,3) | 1(16,7) / 5(83,3) | 3(50) / 3(50) |
| **OSTEOPHYTE** |  | | | | | **0.003** |
| No / Yes | 6(100) / 0(0) | 6(100) / 0(0) | 6(100) / 0(0) | 6(100) / 0(0) | 2(33,3) / 4(66,7) |

*Kruskal Wallis Test, Post Hoc Test: nonparametric post hoc test (Miller 1966) - Pearson Chi Square Test - Linear-by-Linear Association*

*Monte Carlo Simulation results were used in tests*

***^a^*** *Significant relative to Group 1,* **^c^** *Significant relative to Group 3*

*n(%)*

*SC: Small Clones, LC: Large Clones, CT: Cartilage Thickness, BT: Bone Thickness*
